# Report of the Physicians to the Kentucky Penitentiary

**Published:** 1845-01

**Authors:** 


### REPORT OF THE PHYSICIANS TO THE KENTUCKY PENITENTIARY.

The Physicians to the Kentucky Penitentiary, at Frankfort, Drs. W. C. and Lewis Sneed, report one hundred and ninety-six cases of disease as having been treated in that institution during the ten months of their attendance. Of these the most numerous were cholera morbus, intermittent fever, parotitis, and constipation. During all the time not a death has occurred in the penitentiary, although many of the attacks were of a violent character. The physicians conclude their report with the following statement:

"A large number of the convicts sent to this institution are men who have been regular drinkers, or confirmed drunkards, and consequently, enter the prison with constitutions so much impaired by disease and dissipation, that they require more or less medical treatment before they are in a condition for manual labor." Y.

